# Resemblance of Symptoms for Major Depression Assessed at Interview versus from Hospital Record Review

**DOI:** 10.1371/journal.pone.0028734

**Published:** 2012-01-11

**Authors:** Ying Chen, Haimin Li, Yihan Li, Dong Xie, Zhiyang Wang, Fuzhong Yang, Yuan Shen, Sulin Ni, Yan Wei, Yanhua Liu, Lanfen Liu, Chengge Gao, Jun Liu, Lijuan Yan, Gang Wang, Keqing Li, Qiang He, Tiebang Liu, Jinbei Zhang, Yan Ren, Qunli Du, Jing Tian, Honghui Chen, Yanfang Luo, Fengzhi Zhang, Guangwei Sun, Chunjie Shan, Xueyi Wang, Yutang Zhang, Xiaoqin Weng, Yunchun Chen, Zhen Kang, Jing Guan, Yiping Chen, Shenxun Shi, Kenneth S. Kendler, Jonathan Flint, Hong Deng

**Affiliations:** 1 Mental Health Center of West China Hospital of Sichuan University, Chengdu, Sichuan, People's Republic of China; 2 Wellcome Trust Centre for Human Genetics, Oxford, United Kingdom; 3 Fudan University affiliated Huashan Hospital, Shanghai, People's Republic of China; 4 Shanghai Jiao Tong University School of Medicine affiliated Shanghai Mental Health Centre, Shanghai, People's Republic of China; 5 Shanghai Tongji University affiliated Tongji Hospital, Shanghai, People's Republic of China; 6 Nanjing Brain Hospital, Nanjing, Jiangsu, People's Republic of China; 7 No. 4 Affiliated Hospital of Jiangsu University, Zhenjiang, Jiangsu, People's Republic of China; 8 Tianjin Anding Hospital, Tianjin, People's Republic of China; 9 Shandong Mental Health Center, Jinan, Shandong, People's Republic of China; 10 No. 1 Hospital of Medical College of Xian Jiaotong University, Xi'an, Shaanxi, People's Republic of China; 11 No. 1 Hospital of Zhengzhou University, Zhengzhou, Henan, People's Republic of China; 12 No. 1 Mental Health Center Affiliated Harbin Medical University, Harbin, Heilongjiang, People's Republic of China; 13 Beijing Anding Hospital, Capital Medical University, Beijing, People's Republic of China; 14 Hebei Mental Health Center, Baoding, Hebei, People's Republic of China; 15 Shengjing Hospital of China Medical University, Shenyang, Liaoning, People's Republic of China; 16 Shenzhen Kangning Hospital, Luo Hu, Shenzhen, Guangdong, People's Republic of China; 17 No. 3 Affiliated Hospital of Sun Yat-sen University, Guangzhou, Guangdong, People's Republic of China; 18 No. 1 Hospital of Shanxi Medical University, Taiyuan, Shanxi, People's Republic of China; 19 Mental Hospital of Jiangxi Province, Nanchang, Jiangxi, People's Republic of China; 20 The First Affiliated Hospital of Jinan University, Guangzhou, People's Republic of China; 21 Wuhan Mental Health Center, Wuhan, Hubei, People's Republic of China; 22 No. 3 Hospital of Heilongjiang Province, Beian, Heilongjiang, People's Republic of China; 23 Jilin Brain Hospital, Siping, Jilin, People's Republic of China; 24 The First Hospital of China Medical University, Shenyang, Liaoning, People's Republic of China; 25 Dalian No. 7 People's Hospital & Dalian Mental Health Center, Dalian, Liaoning, People's Republic of China; 26 The First Hospital of Hebei Medical University, Shijiazhuang, Hebei, People's Republic of China; 27 Lanzhou University Second Hospital, Second Clinical Medical College of Lanzhou University, Cui Ying Men, Lanzhou, Gansu, People's Republic of China; 28 Psychiatric Hospital of Henan Province, Xinxiang, Henan, People's Republic of China; 29 The Fourth Military Medical University affiliated Xijing Hospital, Xi'an, Shaanxi, People's Republic of China; 30 No. 4 People's Hospital of Liaocheng, Liaocheng, Shandong, People's Republic of China; 31 Guangzhou Brain Hospital/Guangzhou Psychiatric Hospital, Guangzhou, Guangdong, People's Republic of China; 32 Clinical Trial Service Unit, Richard Doll Building, Oxford, United Kingdom; 33 Virginia Commonwealth University, Department of Psychiatry, Virginia Institute for Psychiatric and Behavioral Genetics, Richmond, Virginia, United States of America; Federal University of Rio de Janeiro, Brazil

## Abstract

**Background:**

Diagnostic information for psychiatric research often depends on both clinical interviews and medical records. Although discrepancies between these two sources are well known, there have been few studies into the degree and origins of inconsistencies.

**Principal findings:**

We compared data from structured interviews and medical records on 1,970 Han Chinese women with recurrent DSM-IV major depression (MD). Correlations were high for age at onset of MD (0.93) and number of episodes (0.70), intermediate for family history (+0.62) and duration of longest episode (+0.43) and variable but generally more modest for individual depressive symptoms (mean kappa = 0.32). Four factors were identified for twelve symptoms from medical records and the same four factors emerged from analysis of structured interviews. Factor congruencies were high but the correlation of factors between interviews and records were modest (i.e. +0.2 to +0.4).

**Conclusions:**

Structured interviews and medical records are highly concordant for age of onset, and the number and length of episodes, but agree more modestly for individual symptoms and symptom factors. The modesty of these correlations probably arises from multiple factors including i) inconsistency in the definition of the worst episode, ii) inaccuracies in self-report and iii) difficulties in coding medical records where symptoms were recorded solely for clinical purposes.

## Introduction

Accurate clinical diagnosis is a cornerstone of psychiatric research. Many epidemiological findings of importance for public health, for example those that report the lifetime prevalence and development of psychiatric disorders, often rely on data obtained by interviewing subjects. Clinical data for the majority of these and other research projects are typically collected using a structured interview such as the composite international diagnostic interview (CIDI) or structured clinical interview for DSM (SCID) [1]. Lifetime diagnoses based on structured interviews have good inter-rater reliability [2] but they suffer from a number of limitations.

Foremost among these is that structured clinical interviews assessing a lifetime history of illness rely solely on the accuracy of the subject's memory, which is often imprecise and potentially biased. A number of studies have shown that the reliability of the lifetime prevalence of psychiatric disorders assessed by structured interview is often modest, and that an individual's present mood state impacts on the probability that they will recall a prior depressive episode [3], [4], [5].

There are however alternative sources of information that can be used to augment information from the clinical interview. Medical records provide summaries, usually taken contemporaneously, of information obtained by medical staff involved in the care of patient. The data typically include summaries of interviews and the results of physical and laboratory examinations, together with diagnoses, treatments, and care plans. Medical records can provide an accurate summary of the course of a disease, often recording important events and symptoms the patients themselves do not recall. However, medical records are unstructured and their quality varies, depending on the skills and diligence of the individual physicians and nurses recording the information [6].

Because of the complementary nature of information gleaned from structured interviews and medical records, some researchers combine both sources of information. For example chart diagnosis may not concur with results of a structured interview such as the SCID [7]. However this raises a number of issues, not the least of which is what to do when the two sources of data contradict each other. Despite the importance of these issues, research into the degree and origins of inconsistency between the two sources of clinical data are scant [7], [8]. We have been unable to find a comparison of the two approaches to the diagnosis of major depression (MD).

Here we used data from 1,970 depressed Chinese women to compare these two assessment methods. Because the patients were given a detailed structured interview, covering known risk factors for depression, as well as subject to a careful chart review, we were able to explore patterns of response that might throw light on the nature of the agreements and disagreements between the two assessment methods.

## Materials and Methods

### Ethics statement

The study protocol was approved centrally by the Ethical Review Board of Oxford University (Oxford Tropical Research Ethics Committee) and the ethics committees in all participating hospitals in China. Major psychotic illness was an exclusion criterion, and the large majority of patients were in remission from illness (seen as out-patients). All interviewers were mental health professionals who are well able to judge decisional capacity. The study posed minimal risk (an interview and saliva sample).

### Study subjects

The data for the present study were drawn from the ongoing China, Oxford and VCU Experimental Research on Genetic Epidemiology (CONVERGE) study of MD. These analyses were based on a total of 1,970 cases recruited from 53 provincial mental health centres and psychiatric departments of general medical hospitals in 41 cities in 19 provinces and four central cities: Beijng, Shanghai, Tianjin and Chongqing. All cases were female and had four Han Chinese grandparents. They were aged between 30 and 60, had suffered two or more episodes of MD, with the first episode occurring between the ages of 14 and 50 and had not abused drugs or alcohol before their first episode of MD. Cases were excluded if they had a pre-existing history of bipolar disorder, any type of psychosis or mental retardation.

All cases were interviewed using a computerised assessment system, which lasted on average two hours. All interviewers were mental health professionals, largely psychiatrists and a few psychiatric nurses, trained by the CONVERGE team for a minimum of one week in the use of the interview. The interview includes assessment of psychopathology, demographic and personal characteristics, and psychosocial functioning. Interviews were tape-recorded and a proportion of them were listened to by the trained editors who provided feedback on the quality of the interviews. The interview was semi-structured and required the interviewers to make a range of judgements about the nature and meaning of the reported symptoms. The section of the interview that assessed major depression was adapted from the Composite International Diagnostic Interview (CIDI) (WHO lifetime version 2.1; Chinese version) and classified diagnoses according to the Diagnostic and Statistical Manual of Mental Disorders (DSM-IV) criteria [14].

Additional information using instruments employed from VATSPSUD, was translated and reviewed for accuracy by members of the CONVERGE team. Information on postnatal depression was assessed using an adaptation of the Edinburgh Scale [15]. The history of lifetime major depression in the parents and siblings was assessed using the Family History Research Diagnostic criteria [16]. All available medical records (n = 1,880) were reviewed typically before but sometimes after the interview with the respondent. As would be expected, the quantity and quality of medical records varied considerably. Some included only out-patient records while others included material from in-patient hospitalizations. Notes by treating psychiatrists were almost always available. Notes from nursing staff or other mental health professionals were sometimes included. Interviewers were trained in the completion of the Case Record Rating Scale. If records were available from multiple episodes of illness, they were instructed to focus on the worst episode. They were also instructed to focus on admission and discharge summaries as likely to contain the most complete clinical descriptions. All available case notes were assessed to obtain a diagnosis of DSM-IV depression. The presence of each of 12 symptoms was evaluated as either “clear evidence absent”, “inferred absent”, “inferred present”, “clearly present moderate”, “clearly present severe” or “no information”. Interviewers were also instructed that information obtained from the medical records should never influence their interview of the respondents as the two sources of data were to be kept entirely separate.

The case interview was fully computerized into a bilingual system of Mandarin and English developed in house in Oxford, and called SysQ. Skip patterns were built into SysQ. Interviews were administered by trained interviewers and entered offline in real time onto SysQ, which was installed in the laptops. Once an interview was completed, a backup file containing all the previously entered interview data could be generated with database compatible format. The backup file, together with an audio recording of the entire interview, was uploaded to a designated server currently maintained in Beijing by a service provider. All the uploaded files in the Beijing server were then transferred to an Oxford server quarterly.

### Statistical analysis

Statistical analyses were performed using the software package SPSS 17.0 (SPSS Inc., Chicago, IL). Pearson correlation coefficients were calculated to compare the age of onset of the first episode of depression, the longest duration episode of depression, and the number of episodes. Agreement between the interview and hospital records for 12 individual MD symptoms was assessed by Cohen's kappa statistic [17]. Symptoms were subject to factor analysis with a Varimax rotation again using SPSS software.

## Results

**Figure 1:**
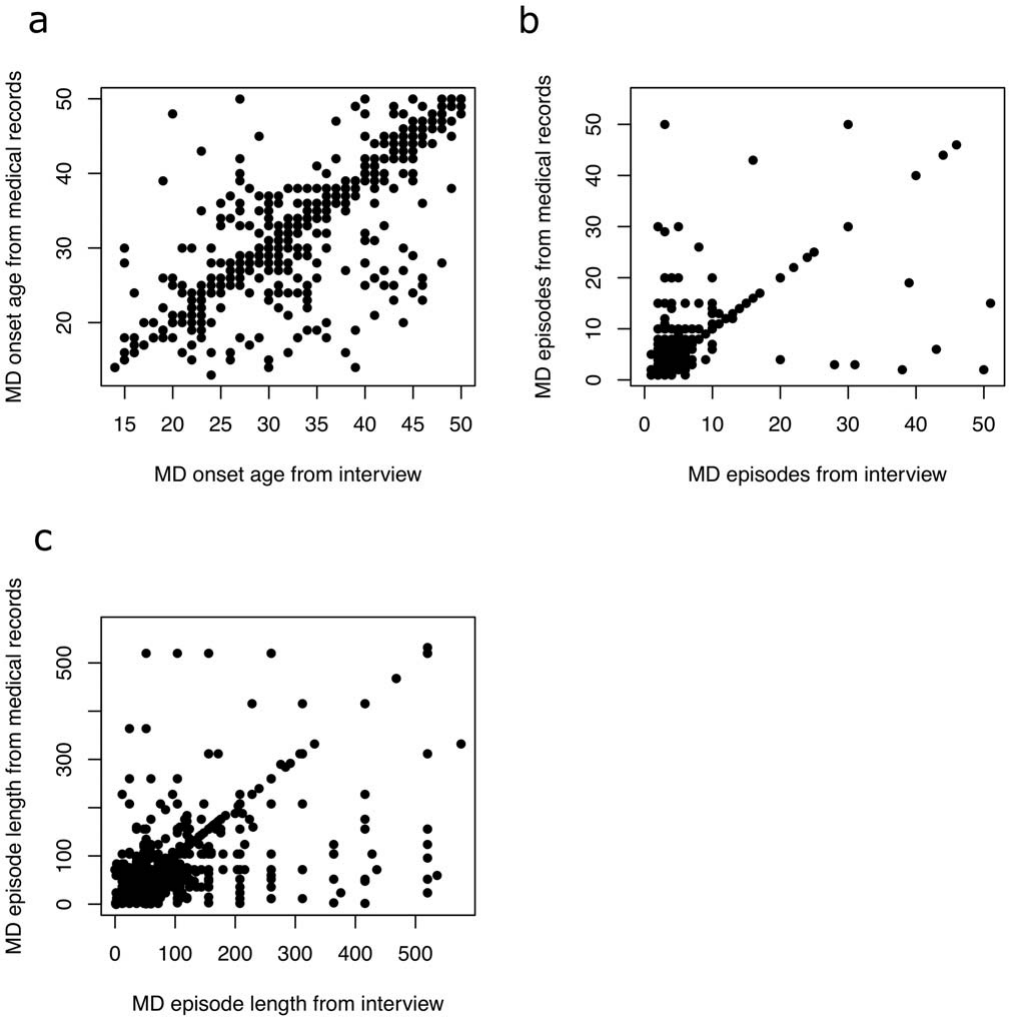
The relationship between information obtained from interviews and medical records for three features of major depression. Each graph plots data from interview data on the horizontal axis against data from medical records on the vertical axis; a) the age of onset (in years) b) the number of episodes c) the duration of the longest episode of major depression (in weeks).

We compared information obtained from medical records and structured interviews on MD episode duration, occurrence, family history and symptomatology. Table 1 shows good correlations for the age of onset of MD, number of episodes and the length of the longest episode (in weeks), with a strikingly high correlation for the age of onset (+0.93). Figure 1 displays graphically the nature of the correlations. Table 2 presents kappa coefficients for family history and for symptoms experienced during the worst MD episode. Family history for depression was relatively reliable across sources (kappa = +0.62). Agreement for symptoms was variable but generally modest (range +0.14 [insomnia] to +0.48 [appetite/weight loss]), with a mean of +0.32. There was no obvious pattern among symptoms that correlated poorly compared to those that correlated more strongly (for example measures of biological symptoms such as changes in sleep and weight varied as much as measures of psychological state, such as feelings of fatigue and thoughts of suicide).

**Table 1:** Correlation between information obtained from a structured interview and medical records for major depression onset and episodes.

	Medical record	Structured interview	N	r	icc
Age of onset	35.3±9.1(14∼50)	34.8±9.3(14∼50)	1693	+0.93	+0.92
Longest duration	80.5±124.7(2∼780)	47.3±84.6(2∼832)	1832	+0.65	+0.65
Number of episodes	3.7±4.3(1∼63)	4.2±5.0(1∼63)	1734	+0.70	+0.67

The table gives the mean, the standard deviation and the range for age in years of onset of major depression, the duration of the longest episode (in weeks) and the total number of episodes. The final two columns give the total number of observations (n), the Pearson correlation coefficient (r), and the intraclass correlation coefficient (icc).

**Table 2:** Correlation between clinical features of major depression obtained from a structured interview and medical records.

	Medical records*		Interview		Kappa
	Yes(%)	No(%)	Yes(%)	No(%)	
Family history	409 (20.8)	1482 (75.2)	630 (32.0)	1287 (65.3)	+0.62
Fatigue/loss of energy	1796 (91.2)	55 (2.8)	1797 (91.2)	138 (7.0)	+0.17
Appetite/weight loss	1351 (68.6)	275 (14.0)	1682 (85.4)	253 (12.8)	+0.48
Appetite/weight gain	175 (8.9)	1196 (60.7)	224 (11.4)	1711 (86.9)	+0.37
Insomnia	1771 (89.9)	75 (3.8)	1781 (90.7)	148 (7.5)	+0.14
Hypersomnia	203 (10.3)	1201 (61.0)	251 (12.7)	1684 (85.5)	+0.40
Psychomotor retardation	1169 (59.3)	393 (19.9)	1434 (72.8)	501 (25.4)	+0.21
Psychomotor agitation	1280 (65.0)	288 (14.6)	1400 (71.1)	535 (27.2)	+0.24
Difficulty concentrating	1543 (78.3)	137 (7.0)	1742 (88.4)	193 (9.8)	+0.18
Suicidal ideation/acts	1342 (68.1)	310 (15.7)	1423 (72.2)	405 (20.6)	+0.41
Irritable/angry	1266 (64.3)	354 (18.0)	1416 (71.9)	514 (26.1)	+0.31
Crying a lot	1230 (62.4)	400 (20.3)	1262 (64.1)	667 (33.9)	+0.46
Nervous/jittery/anxious	1695 (86.0)	142 (7.2)	1730 (87.8)	200 (10.2)	+0.20
Feelings interfere with daily tasks	1767 (89.7)	119 (6.0)	1616 (82.0)	309 (15.7)	+0.40

Kappa is Cohen's kappa statistic (11), all values of which are significant at P<0.0001.

*Sample size varies because of missing data from medical records.

The modest correlation in symptomatology might reflect errors in the way symptoms were elicited and recorded or, since medical records may contain information collected at a different time from the structured interview, it might reflect real differences in the symptom profile of the disease. If the differences were due to random error, then the relationship between individual symptoms might also be disturbed. We tested this by assessing the similarity in factor structure of the symptoms obtained from medical records and structured interview.

We identified four factors from 12 symptoms obtained from medical records with eigenvalues greater than 1. These factors explained 49% of the variance. The first loaded most strongly on three symptoms (psychomotor agitation, irritable/angry, nervous/jittery/anxious symptoms); we label this factor “Anxiety”. The second loaded most strongly on four symptoms (fatigue/loss of energy, appetite/weight loss, psychomotor retardation, and difficulty concentrating); we label this factor “Fatigue”. The third loaded on four symptoms (hypersomnia, insomnia, appetite/weight gain, appetite/weight loss); we label this factor “Neurovegetative”; the last factor loaded heavily on just two symptoms (suicidal ideation/acts, crying a lot), and we have labeled this “Suicide”. Results for the factor analysis of medical records are shown in Table 3.

**Table 3:** Factor Loadings obtained from analysis of 12 major depression symptoms from medical records.

Symptom	Anxiety	Fatigue	Neurovegetative	Suicide
Nervous/jittery/anxious	0.745	0.009	−0.077	−0.037
Irritable/angry	0.620	−0.082	0.043	0.388
Psychomotor agitation	0.606	0.377	0.003	−0.067
Psychomotor retardation	−0.035	0.681	0.156	0.040
Fatigue/loss of energy	0.131	0.568	−0.050	0.000
Difficulty concentrating	0.378	0.487	0.018	0.167
Appetite/weight loss	−0.252	0.467	−0.326	0.262
Hypersomnia	−0.017	0186	0.710	0.033
Insomnia	0.027	0.070	−0.639	0.218
Appetite/weight gain	−0.020	−0.072	0.598	0.288
Crying a lot	0.118	0.035	0.056	0.732
Suicidal ideation/acts	−0.016	0.119	−0.036	0.619

The same four factors emerged from an analysis of structured interviews, explaining 40% of the variance, with Table 4 showing similar loadings to the factors obtained from medical records. Factor congruences (cosines of pairs of vectors defined by the loadings matrix) for the factors extracted from the interviews and medical records were quite high: +0.91 for the Anxiety factor, +0.99 for Fatigue, +0.95 for Neurovegetative and +0.86 for Suicide.

**Table 4:** Factor loadings obtained from analysis of 12 major depression symptoms from structured interviews.

Symptom	Anxiety	Fatigue	Neurovegetative	Suicide
Nervous/jittery/anxious	0.675	0.007	0.028	0.064
Irritable/angry	0.585	−0.104	0.082	0.399
Psychomotor agitation	0.519	0.356	−0.094	−0.028
Psychomotor retardation	−0.008	0.720	0.087	0.032
Fatigue/loss of energy	0.049	0.541	−0.030	0.088
Difficulty concentrating	0.374	0.478	−0.030	−0.108
Appetite/weight loss	−0.161	0.436	−0.544	0.258
Hypersomnia	−0.241	0.217	0.652	0.133
Insomnia	0.343	0.187	−0.453	−0.138
Appetite/weight gain	0.231	0.037	0.665	−0.051
Crying a lot	0.015	0.076	−0.071	0.776
Suicidal ideation/acts	0.095	0.219	0.089	0.529

Finally, we tested the correlation between the factors extracted from chart review and from the structured interviews. Table 5 shows significant correlations between factors obtained from the two sources that are modest to moderate (∼+0.20–0.45). However, the within factor correlations between the interview and medical records are much higher than cross factor correlations (means of 0.27 and 0.04 respectively).

**Table 5:** Correlation between MD Symptom Factors Derived from Personal Interview And Medical Records.

Medical record factor	Interview factor inter-correlation.							
	Anxiety		Fatigue		Neurovegetative		Suicide	
	r	*P*	r	*p*	r	*p*	r	*p*
Anxiety	**0.252**	0.000	0.049	0.063	−0.007	0.790	0.070	0.007
Fatigue	0.003	0.899	**0.225**	0.000	0.013	0.649	0.048	0.059
Neurovegetative	0.058	0.048	0.127	0.000	**0.193**	0.000	0.063	0.015
Suicide	0.048	0.072	0.004	0.879	0.024	0.405	**0.436**	0.000

The correlation (r) and associated p-value (p) between four factors derived from factor analysis of medical records and interview data. The correlation for the same factor is depicted in **bold**.

## Discussion

Our results show that clinical information about MD obtained from unstructured medical records correlates with that from structured clinical interviews, with the degree of correlation depending on the nature of the information. For age of onset, the number and length of episodes and family history, the two sources are highly concordant, but we find variable and generally more modest agreement between individual symptoms. We find the same factor structure present in symptoms from both sources and factor congruence is greater than 0.85 in all cases. This indicates that symptom score differences are unlikely to be due to random error, and that we need to find additional explanations for the discrepancy.

In our study, information from medical records was not contemporaneous with that obtained by interview. This suggests two important reasons for the lack of concordance between symptoms elicited at interview and from medical records. The first is that the information from the two sources may often relate to different episodes of MD. We have no way of knowing whether the worst episode described by the patient corresponds to the worst episode that was picked for rating using hospital records. Consistency of symptoms between depressive episodes is typically modest. For example, the mean correlation between MD symptoms elicited from a large population-based sample of female twins interviewed twice at least one year apart was 0.28 [9]. Similarly modest correlations were found in a study of 78 hospital inpatients examined at intervals of one and two years apart [10]. Both studies found the highest correlations for suicidal behaviour. In our study the correlation between the suicide factors in the two sources of information was also highest (+0.44).

Difference in remembrance is a second reason for discrepancies between the symptoms we acquired from hospital records and from interviews [3], [4], [5]. When subjects are assessed longitudinally recall is known to affect the results, sometimes in predictable ways: for example Bromet et al found that on a second assessment about twice as many patients reported fewer lifetime depressive episodes than those who reported more [3]. However consistency in recall of some items is high, for example reporting of age at onset rarely differs by more than one year [5], a finding that we corroborated.

Psychiatry's reliance on interviews, rather than objective tests, for basic clinical information has spawned a large literature on the reliability of structured ways of assessing patients [1], [11], [12], and typically additional information from chart review is incorporated to obtain a best estimate of lifetime psychiatric diagnosis [2]. These studies show that diagnoses based on interview data alone are an adequate substitute for best estimate diagnoses based on all available data. However there has been much less interest in the validity of chart-derived information. Very few studies have examined the relationship between chart and interview-derived information.

Our results can be usefully compared with a recent study of schizophrenia using a very similar methodology to the current report [8]. Ratings of psychotic symptoms were compared in 1,021 patients with schizophrenia studied in Ireland between personal interview and a review of medical records. Correlations for 21 signs and symptoms of psychotic illness ranged from +0.02 (somatic hallucinations) to +0.55 (religious delusions), with a mean of +0.26. Despite examining a different disease and in a different country, these results are quite similar to those obtained in this study.

A few other studies are moderately relevant to our findings. For example, one study of community practitioners reported a kappa of 0.24 for reliability between chart diagnosis and that obtained from a SCID [7]; by contrast, a survey of diagnoses made on 101 psychiatric inpatients reported high concordance, with most errors judged to have occurred in the charts [13]. It is noteworthy that our findings, pointing to a relatively good agreement in the sources of information, were also obtained from psychiatric hospitals, rather than community physicians, and suggests that the setting in which medical records are obtained may be an important determinant of the reliability of the information.

Our results do not allow us to decide what to do when medical records and interview data disagree. This is most likely to be true for reports of symptoms. While we have shown that there is consistency in factor structure between the two sources of information we have no way of determining the more accurate measure.

Our results should be interpreted in the context of four potentially important methodological limitations. First, an important concern is that medical staff rated the medical records on the same patients that they interviewed. Although interviewers were instructed to keep the two sources of information separate, this may not always have been possible. Therefore we may be overestimating the degree of concordance between medical records and interview-acquired data. Second, the sample is entirely female and our results may or may not extrapolate to men in China. Third, medical records were missing on a small number of cases (n = 90) and often did not contain information about the presence or absence of some individual depressive symptoms. This may introduce an unacknowledged source of bias into our results. Fourth, we have no data on the reliability of our interviews. While we assume that the quality of our interview data is comparable to that of other studies [11] without analysis of repeat interviews we cannot be certain on this point.
